# A framework for population health observatory from a global perspective: proposed preliminary maturity model and key performance indicators

**DOI:** 10.3389/fpubh.2026.1901078

**Published:** 2026-09-15

**Authors:** Mariam M. AlEissa, Shaker A. Alomary, Abdullah M. Assiri

**Affiliations:** 1 College of Medicine, Alfaisal University, Riyadh, Saudi Arabia; 2 Population Health Observatory, Ministry of Health, Riyadh, Saudi Arabia; 3 Public Health Lab, Public Health Authority, Riyadh, Saudi Arabia; 4 King Khaled Eye Specialist Hospital (KKESH) Research Centre, Riyadh, Saudi Arabia; 5 King Abdullah Petroleum Studies and Research Center (KAPSARC), Riyadh, Saudi Arabia

**Keywords:** artificial intelligence, comparative health policy, digital health, health intelligence, intervention, KPIs, population health, population health observatory

## Abstract

**Background:**

Population health observatories (PHOs) have emerged as fundamental pillars of modern public health governance, enabling the transformation of fragmented, multidimensional health data from multiple centres into evidence-informed policymaking and development, strategic planning and actionable intelligence for surveillance and precision public health. This study aimed to develop a globally comparative framework for assessing the maturity level of PHOs by using standardised artificial intelligence (AI) and a series of multidimensional key performance indicators (KPIs), with particular emphasis on the Saudi Arabian healthcare transformation context.

**Methodology:**

We conducted a structured document review and qualitative comparative assessment of national PHOs or equivalent health intelligence systems in 21 jurisdictions. Evidence was obtained from peer-reviewed literature, governmental publications, international agency reports and publicly accessible national documentation. Each jurisdiction was assessed using 18 predefined variables covering data infrastructure, interoperability, surveillance, analytics, governance and population. The resulting scores were mapped to a proposed five-level maturity continuum, and candidate KPIs were organised across seven functional domains.

**Results:**

This study demonstrated substantial international heterogeneity in PHO AI maturity, predominantly driven by the variables of interoperability of data, real-time surveillance capabilities, deployment of predictive analytics, integration with registry ecosystems and implementation of AI governance. The United Kingdom, the Netherlands, Finland, South Korea and Singapore exhibited maturity levels ranging from systemic to nearly autonomous. Concurrently, Saudi Arabia was positioned at operational maturity Level 3, reflecting substantial advancement in digital transformation, early AI implementation and registry integration. At the same time, gaps persist in terms of wearable and Internet of Things (IoT)-derived data integration, genomic integration, biobank interoperability and autonomous decision support infrastructure.

**Conclusion:**

The preliminary framework identified variations in documented PHO capabilities and may support structured gap assessment. Formal external validation and prospective testing are required before this framework can be used for national benchmarking or evaluation of population health impacts. These steps are essential for strategic priority setting in Saudi Arabia’s PHO as the country advances towards Level 5 autonomous maturity through robust AI governance, interoperable health ecosystems, predictive and prescriptive analytics and precision public health integration. Such a transformation could position Saudi Arabia as a regional and global leader in precision public health aligned with Vision 2030.

## 1 Introduction

Population health observatories (PHOs) are components of a regulatory infrastructure that plays a critical role in public health services through the gathering, analysis and distribution of health-related data. PHOs play a particularly important role in disease surveillance and strategic planning to improve population health outcomes (1). The evolution of the PHO aligns with the global imperatives towards sustainable development goals, universal health coverage and the promotion of health equity. The PHO emerged as a pivotal pillar of data-driven governance within contemporary health systems, acting as a cornerstone of strategic decision making and policy formulation (2). With rapid progress in artificial intelligence (AI), interoperable data ecosystems and digital health infrastructures, the scope and functionality of PHOs have been profoundly redefined in the past two decades. In leading jurisdictions, such as the United Kingdom (UK) (3–5), Canada (6, 7), and the Netherlands (8–11), these platforms have evolved well beyond traditional descriptive epidemiology reporting systems. They now serve as sophisticated engines for the optimisation of health resource allocation, predictive modelling and anticipatory public health intelligence, thereby empowering proactive, precision-informed governance (3–5, 9–13).

This advancement requires rigorous and systematic deployment of key performance indicators (KPIs) for AI maturity, which constitute a fundamental aspect of this public health transformation by enabling the assurance of quality standards, the precise measurement of progress and the facilitation of robust benchmarking across comparable domains (8).

Although the need for integrated health intelligence systems is global, individual jurisdictions differ considerably in governance, digital infrastructure, surveillance capability, analytical readiness and access to population-level data. A comparative framework may therefore help describe these differences and identify common capability gaps without assuming that one national model is universally applicable. Saudi Arabia was included in this study as an illustrative case within a broader international comparison. Its ongoing digital health transformation, establishment of a national PHO and policy emphasis on data-driven prevention provide the context in which to explore how the proposed framework might support structured gap identification and future planning.

In Saudi Arabia, the Ministry of Health’s establishment of a national PHO reflects a deliberate strategic convergence with the ambitions of Saudi Vision 2030 by accelerating digital transformation, advancing health system modernisation, increasing life expectancy, encouraging innovation in healthcare and institutionalising evidence-based policymaking (3, 7, 14, 15). Within this context of modernisation in Saudi Arabia, marked regional health disparities represented by the epidemiological burden have presented a high prevalence of noncommunicable diseases (NCDs) and inherited genetic diseases (16–18). Consequently, a resilient and comprehensive PHO infrastructure is not merely a privilege but an imperative to serve as a platform for real-time surveillance of critical diseases and to enable sophisticated risk stratification and personalised interventions at the population level (19). However, the ecosystem of PHOs in Saudi Arabia remains in its formative stage, with current capabilities positioned along a continuum between experimental and operational in terms of AI maturity (14, 16, 20, 21). This calls for a transitional posture that requires a rigorously structured framework to define performance expectations that can be clearly articulated, precisely identify gaps and strategically optimise resource allocation (22, 23). Evidence drawn from international experiences shows that deliberate formulation of KPIs across domains, such as governance, data quality, stakeholder engagement and the translation of research into policy and practice, enhances overall system accountability, transparency and effectiveness (14, 24, 25). The integration of AI within PHOs introduces a nuanced dimension of complexity and opportunity.

AI offers advanced capabilities in supporting the detection of disease outbreaks, modelling for risk prediction and health system simulation (26, 27). However, its deployment and implementation necessitate rigorous oversight to ensure adherence to principles of accuracy, analytical validity, explainability and algorithmic transparency (28). To maintain institutional capability and governance and provide important foundations for evaluating infrastructure, it is essential to create AI readiness frameworks, health information system assessments and digital health maturity models (29, 30). The PHO-specific structure requires multiple layers of function integration within a single assessment, including multisource data linkage, research translation, population surveillance, epidemiological intelligence, equity monitoring and the conversion of evidence into policy action. Any future proposed model will not replace existing frameworks (1, 31). Therefore, the framework should be interpreted as a proposed integrative model requiring psychometric evaluation, formal stakeholder consultation and external validation.

Accordingly, the primary research question was: To what extent can a proposed multidimensional maturity framework differentiate the documented governance, data integration, surveillance and analytical and policy translation capabilities of selected national PHOs or equivalent health intelligence systems?

The primary objective of this study was to develop and apply a preliminary multidimensional maturity framework for the comparative assessment of national PHOs. The secondary objectives were to (1) identify cross-national patterns in documented PHO capability, (2) explore the applicability of the framework across jurisdictions at different stages of digital health development, and (3) demonstrate its application through an illustrative case study of Saudi Arabia. This study was designed as a descriptive comparative framework development study and did not evaluate whether higher maturity scores are associated with improved population health outcomes.

## 2 Materials and methods

### 2.1 Study design

The study involved a structured document review, conducting a qualitative comparative analysis between October 2025 and May 2026 and utilising narrative literature, peer-reviewed sources, official government documents, international agency publications and PHO and government reports. To expand the search, we used Elevare PRISM Version 1.0 (Elevare Partners, Riyadh, Saudi Arabia, https://www.elevare360advisory.com) as a supplementary research discovery tool for document retrieval and literature identification (2026). The research engine was limited to search identification and augmentation of potentially relevant sources. It was not used for paper extraction, maturity classification, scoring, analysis, interpretation of results or generation of conclusions. The platform uses a proprietary search and ranking process; its algorithmic operation, coverage and ranking performance could not be independently evaluated. Potential limitations therefore included incomplete retrieval, ranking bias, limited transparency and dependence on indexed online content. All included documents were assessed and reviewed by the research team using the predefined maturity framework. Data were gathered from 21 countries: Saudi Arabia, the UK, Canada, the United States (US), the Netherlands, Sweden, Finland, France, Singapore, Japan, South Korea, India, Chile, Cameroon, Brazil, Spain, Iran, Mexico, Colombia, South Africa and Sub-Saharan Africa (see Supplementary Table 1). The countries were selected based on the availability of publicly accessible national health intelligence platforms and public documentation, such as observatory reports, digital health strategies and AI implementation documentation, for an existing equivalent health intelligence body or national PHO in high- and middle-income countries. In addition, eligibility required sufficient publicly accessible information to permit the assessment of several framework domains. Consequently, the sample was not intended to represent all countries or to generate definitive global rankings. Because public documentation varies substantially between jurisdictions, each country assessment was assigned an evidence confidence rating based on source completeness, recency, independence and consistency.

Sub-Saharan Africa was included as a single regional unit rather than as separate countries because the study’s unit of analysis was the existing PHO or equivalent health intelligence body itself, not the country as an administrative unit. Countries such as Cameroon and South Africa were included individually because each maintains its own distinct national PHO/observatory, run by a distinct national institution, meeting the eligibility criteria independently. For the remainder of Sub-Saharan Africa, however, no comparable distinct national PHOs met the study’s eligibility criteria; the identifiable, documented and publicly assessable observatory infrastructure at this level of maturity was the regional African Health Observatory, which functions as a shared, multi-country platform rather than a set of independent national systems. Consequently, Sub-Saharan Africa was scored as a single regional entity to reflect the actual institutional structure of the observatory that exists and was assessed, rather than artificially disaggregating a shared regional platform into individual country scores for which no distinct national-level evidence existed.

All documents were manually reviewed by the investigators, and the data were extracted using a standardised assessment matrix (Supplementary Table 2). Information was coded according to qualitative to quantitative numbers based on predefined variables (see Supplementary Table 3). Assessments at the country level were independently reviewed by the research team using a predefined 18-variable assessment. Before final scoring, any discrepancies were resolved through a consensus review, and assigned scores were discussed among the investigators. A calibration exercise was performed to harmonise the interpretation of scoring to ensure consistent definitions across the evaluated domains. The maturity final scores represented agreed-upon values following the reconciliation of all scoring differences. Microsoft Excel was utilised for data extraction, scoring, normalisation and the generation of comparative visualisations, including a global heat map. Countries were compared through the AI maturity and KPI framework by establishing certain models and standards to evaluate them, as described below.

### 2.2 Data sources and comparative document review

This study employed a comparative document review rather than a systematic review. Information was collected between October 2025 and May 2026 from publicly available official sources, including national Ministries of Health, PHO websites, government public health agencies, World Health Organization (WHO) publications, Organisation for Economic Co-operation and Development (OECD) reports, digital health strategies and other official governmental documents. Peer-reviewed literature was consulted where appropriate to provide contextual information and support the interpretation of national approaches.

Twenty-one countries were purposively selected to represent diverse geographical regions and varying levels of public health and digital health maturity. For each country, standardised information was extracted regarding governance, surveillance systems, interoperability, analytical capability, artificial intelligence implementation, genomics integration, data infrastructure and policy translation. Documents were included when they originated from official governmental or internationally recognised organisations and contained sufficient information to evaluate one or more components of the proposed PHO maturity framework. Information from all countries was extracted using the same predefined assessment framework to ensure consistency across comparisons.

### 2.3 Framework development process for assessing digital health maturity

A structured conceptual mapping approach was considered for the development of PHO frameworks to assess digital health maturity. These frameworks were generated through narrative synthesis of existing international frameworks, including WHO digital health maturity guidance, OECD AI governance principles and the combined Learning Health System and Donabedian models supplemented by investigator consensus, rather than through a systematic identification process, a formal Delphi exercise, structured stakeholder consultation or a quantitative content validity assessment. This process resulted in 18 candidate maturity variables covering descriptive reporting, AI implementation, clinical-data integration, real-time data exchange, registry linkage, interoperability, biobanks, genomics, social and environmental determinants, wearable data, predictive and prescriptive analytics, resource allocation, autonomous systems, automation, population coverage and AI governance. These variables were categorised into seven KPIs covering the structure, process and outcome. Given this, the framework should be regarded as a preliminary face-valid model rather than as a psychometrically validated instrument. Future work should subject these variables and domains to formal content-validity testing (e.g., expert panel rating with content validity indices), a Delphi consensus process involving PHO stakeholders across countries and construct/criterion validation against independent maturity benchmarks before the framework is adopted for formal cross-national benchmarking or policy decision making (32–35).

### 2.4 Scoring rules and evidence requirements

Each variable was scored on an ordinal scale from 0 to 5 using predefined evidence anchors: 0 indicated that no evidence of capability was identified; 1 indicated planning or isolated preliminary activity; 2 indicated an early pilot or geographically restricted implementation; 3 indicated established implementation in multiple organisations or regions without comprehensive national integration; 4 indicated broad national implementation with documented governance and routine use; and 5 indicated nationally integrated, routinely monitored and outcome-linked implementation. A score was assigned only when supported by at least one identifiable official source or two concordant independent sources. When evidence was conflicting, the lower defensible score was used. When no publicly accessible evidence was found, the item was coded as ‘not verifiable’ rather than automatically interpreted as an absence of capability.

The six-point ordinal anchors (0–5) were designed for cross-country comparability and evidentiary transparency, not to capture fine-grained variations in scale, coverage or resourcing within a given band. Countries assigned the same score for a variable met the same minimum evidentiary threshold for that band but could still differ substantially in underlying capacity; the score reflected the documented maturity stage reached, not the magnitude of investment or infrastructure within that stage.

### 2.5 AI maturity model

PHOs universally rely on AI for data analytics and the knowledge that there are standards for measuring AI maturity in health in different countries, set by the WHO (21). In this study, we extrapolated from the WHO AI maturity standards and created a scoring system for AI maturity in which the final score was calculated by averaging the 18 maturity variables and then multiplying by 20 to normalise values on a 0–100 scale. Equal weighting was deliberately adopted at this initial development stage rather than as a default. No validated, empirically derived weighting scheme currently exists linking individual AI/data maturity domains to population health outcomes across public health organisations, and any investigator-assigned differential weights (e.g., prioritising AI governance or interoperability over other domains) would themselves constitute an unvalidated, subjective judgement rather than a more objective alternative. Equal weighting was therefore chosen for methodological transparency and reproducibility. This indicates that every domain is treated as a necessary, non-substitutable component of maturity, and it avoids embedding untested assumptions about relative domain importance into the composite score at a stage where the framework itself has not yet been validated. Furthermore, we categorised the maturity level of AI integration in PHOs according to the final score, which was mapped to the five-level AI maturity classification described in Table 1.

**Table 1:** The AI maturity model for each country was assessed against 18 predefined variables (see Supplementary Tables 2, 3) extracted from the world’s best practices, derived from WHO digital health maturity guidance, OECD AI governance principles and published international observatory frameworks.

Maturity level	Category	Score range	Core definition	Required/Typical variables
Level 1	Foundational	0–20	Static dashboard systems and reports only provide historical descriptive analytics with limited or no machine learning or AI-driven models (Excel, Power BI and Tableau) or any predictive insights. Decision making is human-driven, slow and reactive. Provides annual or quarterly reports of counts, percentages, trends over time, prevalence and incidence rates.	Static dashboards; annual/quarterly reports; no machine learning (ML); no electronic health records (EHR), integration; siloed datasets
Level 2	Experimental	21–40	Provides AI pilots with models in isolated settings, with no automation for testing accuracy and feasibility. The results are premature for real-world decision making due to a lack of prioritisation and foresight.	AI pilots; basic ML feasibility; limited validation; limited interoperability; no national deployment
Level 3	Operational	41–60	AI is embedded in selected workflows and routine data systems.	EHR/registry/lab integration; risk stratification; partial real-time feeds; decision support; partial interoperability
Level 4	Systemic	61–85	The AI supports real-time data embedding into routine workflows, such as EHR registries and laboratory tests, for prompt, measurable impact on care, risk stratification or policy decisions, or clinical lead decision support; furthermore, it prioritises high-risk populations and supports resource allocation.	Full/strong registry linkage; biobank/genomics; social determinants of health (SDOH)/environment; predictive and prescriptive analytics; resource allocation
Level 5	Autonomous	86–100	A nationally integrated PHO that uses interoperable and near-real-time data to support continuously evaluated predictive and prescriptive analytics, rapid public health response and accountable decision support. Automation operates within predefined governance, human oversight and safety requirements. Advanced capabilities such as wearable integration, federated analytics, simulation models or digital twins may be present but are not mandatory for classification at this level.	Wearables/Internet of Things (IoT); real-time streaming; autonomous execution; digital twins; continuous learning systems; personalised prevention

### 2.6 KPI framework

To reach this maturity level, a framework based on a set of KPIs was implemented. We defined seven domains: Governance and Strategic Leadership, Data Integration and Digital Infrastructure, Epidemiology and Surveillance, AI and Predictive Analytics, Research, Innovation and Policy Translation, Preventive Health and Screening Programs and Population Health Outcomes and Equity. These domains were primarily derived from a combination of the two models. The Learning Health System (LHS) model was chosen because it supports the continuous transformation of health data into measurable improvements in population outcomes, such as continuous learning, precision public health and evidence-based policymaking. The Donabedian model was used as a quality assessment approach (36, 37), selected to classify indicators into structure indicators, system capacity and infrastructure, process indicators for operational performance and workflows and outcome indicators to measurable population health impact for systematic evaluation of health system performance. Focusing on standardised domains aligned with modern digital public health systems and Saudi Vision 2030 transformation priorities (37–39), including strategic domains encompassing governance, digital infrastructure, surveillance, analytics, preventive healthcare and population outcomes.

The two conceptual models were used for differing but complementary purposes. The LHS model provided the longitudinal logic of the framework. Population data were collected and analysed, converted into evidence, translated into policy or intervention and subsequently reassessed through outcome feedback. The Donabedian model provided the measurement architecture for each stage by distinguishing the resources and governance required to operate the PHO (structure), the activities performed by the PHO (process) and the resulting policy or population-level effects (outcomes). The seven KPI domains were mapped across both dimensions so that each functional area contained structure, process and outcome indicators.

## 3 Results

### 3.1 Global AI maturity comparison

The variables collected revealed substantial data on AI maturity across PHOs, reflecting differences in digital infrastructure, interoperability and the implementation of wearable/predictive analytics. As indicated in Figure 1 and Supplementary Tables 2, 3, most high-income countries (40) (as classified by the World Bank) demonstrated operational to systemic maturity levels due to established interoperability frameworks, integrated registries and real-time analytical capabilities. In contrast, in low-income countries, the developing systems remained predominantly in the experimental stage. The scoring was evaluated in Supplementary Table 3 according to the availability and efficiency of the integrated AI services.

**Figure 1:**
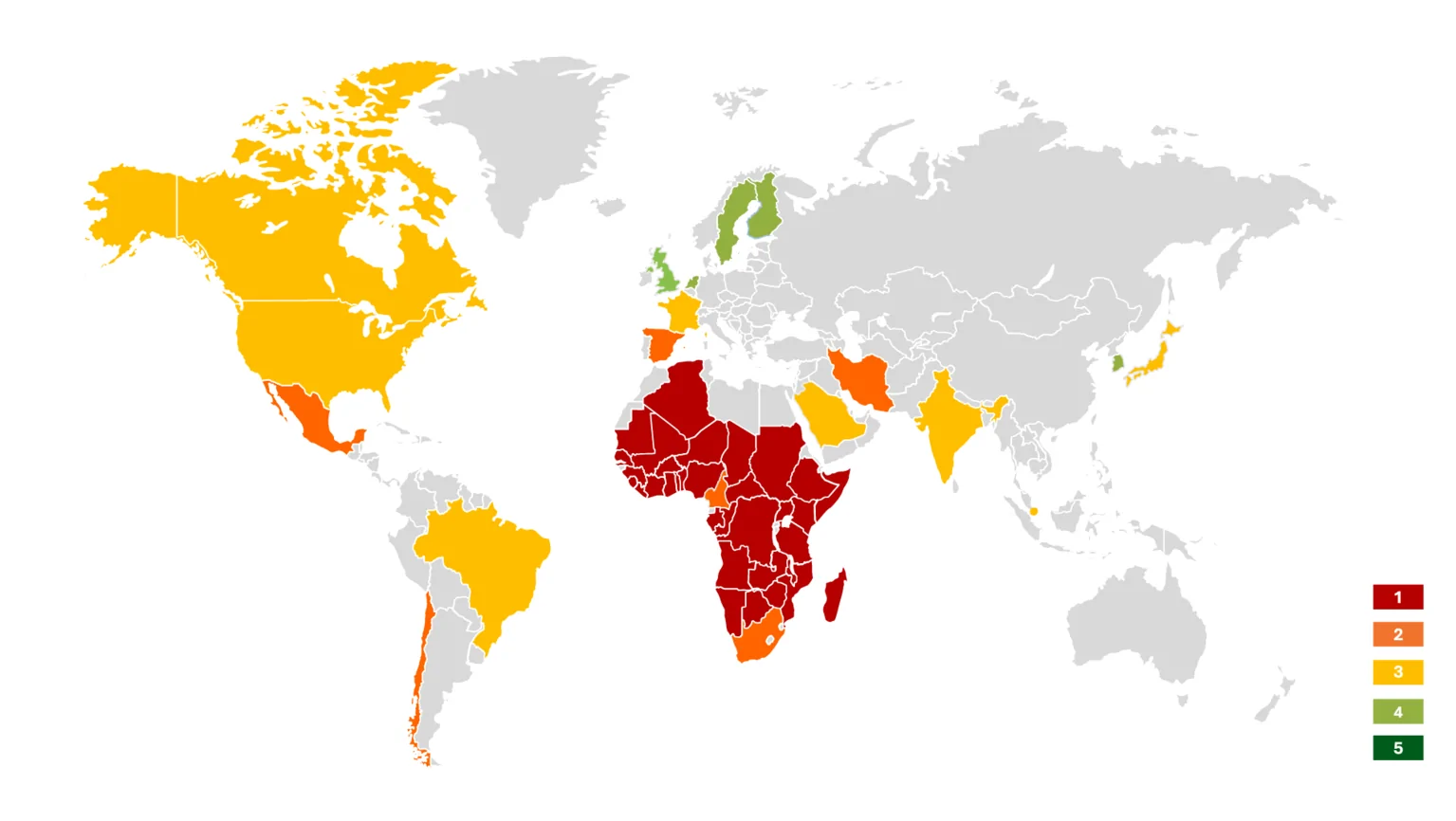
Global heat map showing the comparative artificial intelligence (AI) maturity of PHOs in 21 countries. The global heat map illustrates the relative positioning of national PHOs using a five-level AI maturity scale (Table 1), ranging from foundational (Level 1) to autonomous (Level 5). High-income countries, such as the United Kingdom, Finland, Sweden, the Netherlands, Singapore, and South Korea, demonstrated advanced or near-autonomous AI integration, while middle-income countries and emerging PHOs, including Saudi Arabia, remained in the experimental to operational phases. The levels, which are based on 18 variables of maturity, are indicated in Supplementary Table 2 and Figure 2.

As indicated in Figure 2, the countries that reached high AI maturity, such as the United Kingdom, Finland, Sweden, the Netherlands, Singapore and South Korea, demonstrated substantial heterogeneity that characterises the highest maturity tier, reflecting long-standing investments in integrated data infrastructure, real-time surveillance and policy-embedded analytics. South Korea and Singapore also demonstrated advanced maturity, particularly in digital health intelligence and infectious disease forecasting. Notably, Singapore outperformed the United Kingdom on several individual variables, although both countries reached the highest overall maturity tier. This is consistent with structural differences between the two systems: Singapore’s single, centrally coordinated National Electronic Health Records (NEHR), in place since 2011 and now reinforced by a 2026 Health Information Bill mandating nationwide data contribution from all public and private hospitals, has enabled faster, more uniform integration of AI tools, such as the Next Generation Electronic Medical Record programme across its smaller, unified health system (41–43). In contrast, National Health Service (NHS) AI adoption in the United Kingdom has been documented as fragmented and dependent on local trust-level infrastructure, procurement and governance decisions. Reports have found that AI initiatives in the United Kingdom remain concentrated in isolated pilots rather than in system-wide deployment, even where a national strategy exists (44, 45). Singapore’s smaller population, single-payer/centralised health administration and ‘Smart Nation’ digital governance strategy, implemented in 2014, enable faster nationwide rollout of specific technical capabilities than the UK’s larger, devolved, multi-trust NHS structure, even though both ultimately reach comparable overall maturity classifications. Saudi Arabia is at Level 3, indicating the early institutionalisation of AI pilots with limited systemic integration (Supplementary Table 1).

**Figure 2:**
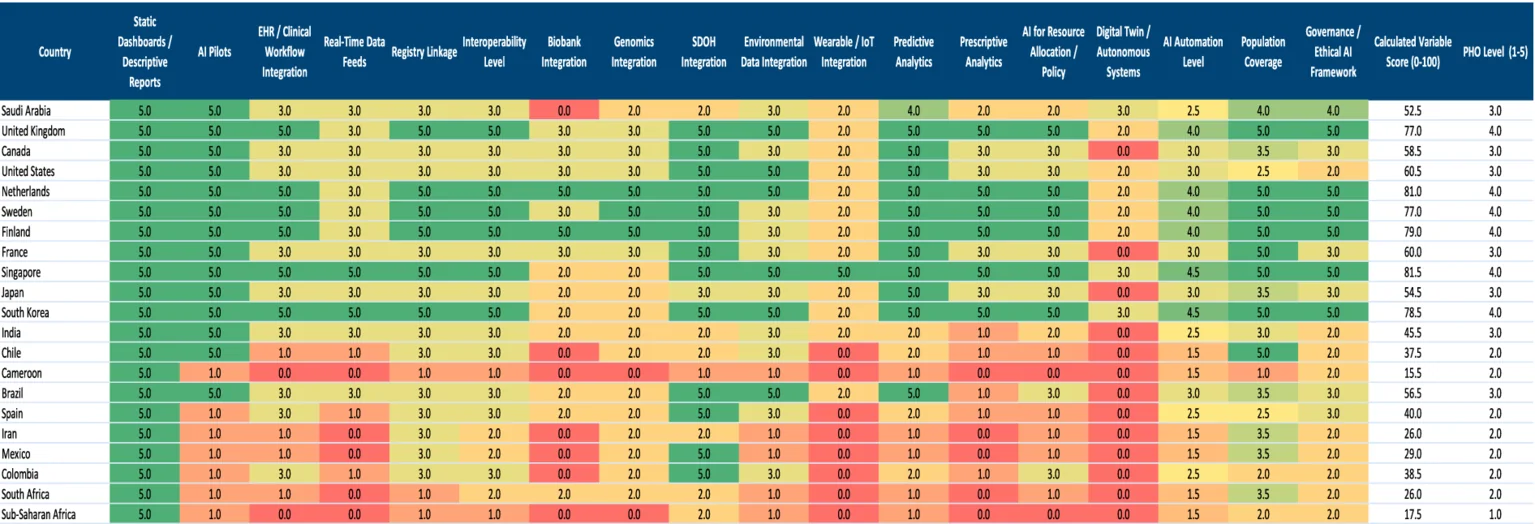
Heat map of the comparative AI maturity and benchmarking framework for PHOs in 21 countries. Higher variable scores (5) indicate stronger performance and institutional maturity.

Three broad patterns emerged from the comparative profiles. First, strong interoperability, registry linkage and governance tended to occur together, suggesting that advanced analytical capability depended on established institutional and data foundations. Second, predictive analytics was more widely documented than prescriptive analytics, autonomous execution or digital-twin capability, indicating that most systems remained decision support platforms rather than autonomous public health systems. Third, genomics, biobank and wearable-data integration varied substantially even among higher-income jurisdictions, suggesting that these capabilities were influenced not only by national income but also by governance structure, research infrastructure, data-sharing policy and the degree of centralisation. Lower-scoring systems were generally characterised by fragmented data sources, limited real-time exchange and restricted implementation beyond pilot settings.

Assessing a PHO’s AI maturity required the application of our proposed KPI framework to establish a comprehensive multidimensional approach for assessing PHOs relative to the governance, surveillance, digital infrastructure, integration of AI, research translation, preventive health and population impact domains. The framework systematically categorised indicators into structure, process and outcome components, thereby enabling standardised benchmarking of PHO maturity, operational readiness and population-level outcomes. Following the Donabedian framework, indicators were categorised into structure, process and health outcome categories, as shown in Table 2. The LHS model further guided the assortment of areas, emphasising continuous evidence translation, data generation and iterative improvement in population health outcomes. This highlights that mature observatories were characterised by strong interoperability, advanced predictive analytics, integrated surveillance systems and effective policy translation mechanisms, whereas developing systems showed limitations in AI governance, real-time data integration and implementation capacity. This framework provided a scalable model for monitoring PHO performance and guiding strategic development towards precision public health and data-driven healthcare transformation.

**Table 2:** KPIs for the evaluation of PHOs relative to the structure, process and outcome indicators.

Indicators	Structure indicators	Process indicators	Outcome indicators
Governance and strategic leadership	National PHO governance framework established, dedicated PHO budget allocation, national AI health strategy and ethical and regulatory framework	Frequency of governance meetings, stakeholder engagement activities and frequency of policy reviews	Policy implementation efficiency, alignment with Vision 2030 and improved institutional accountability
Data integration and digital infrastructure	National electronic health records (NEHR) integration coverage, registry linkage capability, AI-enabled PHO platform and cybersecurity framework	Real-time data exchange capability, automated reporting workflows, data completeness and validation rate	Reduction in reporting delays, improved surveillance accuracy, increased data reliability and interoperability
Epidemiology and surveillance	National disease registries, public health dashboards and genomic surveillance integration	Timeliness of outbreak detection, epidemiological reporting frequency and implementation of population risk stratification	Reduced outbreak response time, earlier disease trend detection and improved preventive targeting
Artificial intelligence and predictive analytics	AI infrastructure availability, AI workforce capacity and AI governance framework	Number of deployed predictive models, AI model validation frequency and use of predictive analytics	Improved forecasting accuracy, reduction in manual workload and optimised resource allocation
Research, innovation, and policy translation	Research-policy integration unit, national public health research collaborations and advanced analytics infrastructure	Number of policy briefs generated, peer-reviewed publications and cross-sector implementation projects	Evidence-informed policymaking, increased research translation into practice and improved healthcare planning
Preventive health and screening programs	Availability of national screening programs, geographic coverage of preventive services and risk-assessment tools	Screening participation rate, referral completion rate and public awareness campaign implementation	Increased early disease detection, reduced late-stage disease presentation and improved preventive behaviours
Population health outcomes and equity	Health equity monitoring systems, SDOH integration and quality-of-life assessment tools	Monitoring frequency of vulnerable populations, high-risk intervention implementation and follow-up compliance rate	Reduction in regional health disparities, reduced disease burden and mortality and improved survival and quality of life

The domain-level indicators above provide the conceptual structure of the KPI framework. Illustrative candidate KPIs are distinguished from externally validated performance standards, and each domain is operationalised with precise definitions, calculation methods, data sources, reporting frequencies and proposed benchmarks in Supplementary Table 4.

The KPIs presented in this study are candidate operational measures rather than validated international performance standards. Each indicator should be adapted to the jurisdiction’s data architecture, baseline capacity and policy priorities. To support implementation, a fully operationalised version of these indicators specifying operational definitions, calculation methods, data sources, reporting frequency and proposed targets is provided in Supplementary Table 4.

### 3.2 Saudi Arabia current positioning

Saudi Arabia’s PHO is currently positioned at Level 3. Based on the primary equal-weight document assessment, Saudi Arabia received an estimated maturity score of 52.5/100, corresponding to Level 3 under the proposed classification. This figure should be interpreted as an exploratory point estimate rather than as a precise or independently verified national measure. Under alternative plausible weighting and scoring assumptions, the estimated score ranged from 41 to 60, as explained in Table 3, while the operational AI maturity category remained Level 3. As indicated in Figures 1, 2, progress in dashboards, AI pilots, registry use, predictive analytics and governance totalled a score of 52.5/100, meaning that AI and data systems are already being used in selected workflows but are not yet fully integrated across the national public health systems. Early-stage biobank initiatives exist, including those at Princess Nourah Bint Abdulrahman University and the Public Health Authority, but remain institutionally restricted rather than nationally coordinated; this is consistent with the study’s early pilot or geographically restricted implementation scoring anchor (46–48). Gaps remain in genomics integration, wearable/IoT data, prescriptive analytics and autonomous decision-support systems, where no comparable pilot-stage evidence was identified.

**Table 3:** Proposed policy considerations arising from the Saudi PHO case assessment to level 5: AI-enabled maturity.

Priority area	Identified capability gap	Proposed action	International implementation basis	Feasibility requirements	Indicative time horizon	Evidence status
Interoperability and data integration	Fragmented EHRs, registries, laboratory systems, and biobanks with no unified national linkage	Link national EHRs, disease registries, laboratories, genomic databases, biobanks, and screening programs through unified interoperable platforms and standards	Singapore’s National Electronic Health Record (NEHR) required approximately a decade of phased national implementation under a single-payer, centrally governed system to achieve nationwide provider linkage (42)	Requires unified data standards, sustained multi-year funding commitment, and centralised governance authority across ministries/institutions	Long-term (8–10 + years, based on comparable single-payer nationwide EHR rollouts)	Illustrative external benchmark; no Saudi-specific costing or feasibility assessment conducted
Predictive and prescriptive analytics	Predominantly descriptive dashboards; limited transition to AI-driven forecasting or resource-allocation systems	Transition from descriptive dashboards to AI-driven predictive and prescriptive systems for forecasting, risk identification, and resource allocation	NHS England’s AI adoption, despite an established national strategy, was documented in 2025 as remaining largely in early pilot phases rather than fully embedded into routine care, even in a well-resourced system (44)	Requires trust/institution-level infrastructure investment, workforce AI literacy, and a system-wide (not pilot-isolated) implementation strategy	Medium- to long-term (multi-year even post-strategy launch, per NHS experience)	Illustrative external benchmark; no Saudi-specific costing or feasibility assessment conducted
Genomics and precision public health integration	Limited integration of genomics, polygenic risk scores, and biobank data into routine PHO infrastructure	Integrate genomics, polygenic risk scores, biobanks, and precision medicine frameworks into PHO infrastructure for inherited disorders and noncommunicable diseases	The UK’s 100,000 Genomes Project ran from 2013 to completion in December 2018 (~5–6 years) before transitioning into the NHS Genomic Medicine Service for routine clinical care (88)	Requires dedicated national sequencing infrastructure, consented biobank cohorts, clinical genomics workforce, and a pathway from research project to routine clinical/public health service	Long-term (5–6 + years from program launch to routine-care integration, per UK experience)	Illustrative external benchmark; no Saudi-specific costing or feasibility assessment conducted
AI governance and trust frameworks	No established national framework for AI ethics, explainability, bias monitoring, or regulatory oversight specific to PHO/public health AI	Establish national frameworks for AI ethics, explainability, bias monitoring, validation, cybersecurity, privacy protection, and regulatory oversight	The EU AI Act phases in high-risk AI system obligations over a staggered ~3-year period, illustrating that comprehensive AI governance frameworks are typically implemented in stages rather than as a single regulatory action (89)	Requires legislative/regulatory drafting capacity, designated national competent authority, conformity-assessment infrastructure, and phased rather than single-step enactment	Medium-term (3 + years for phased regulatory rollout, per EU experience)	Illustrative external benchmark; no Saudi-specific costing or feasibility assessment conducted

### 3.3 Proposed expert-informed policy considerations for the Saudi PHO

The following roadmap is presented as an expert-informed policy scenario derived from the capability gaps identified in the Saudi case assessment and from general international implementation principles. Its phases and timelines were not empirically estimated from the comparative data and should not be interpreted as validated predictions of implementation feasibility. A detailed cost analysis, workforce assessment, legal review, infrastructure analysis and stakeholder consultation would be required before adoption. Saudi Arabia must move from fragmented operational systems to a fully integrated, AI-enabled, real-time public health intelligence ecosystem to reach Level 5. This requires real-time data streams, autonomous execution within approved governance frameworks, digital twins, wearable/IoT integration, continuous learning systems and personalised prevention, as described in Figure 3. In addition, this requires priority improvement areas to be implemented and enforced by the Ministry of Health (MoH), as indicated in Table 3. Furthermore, it will necessitate a personalised set of KPIs to be implemented to ensure strong performance, as illustrated in Figure 4.

**Figure 3:**
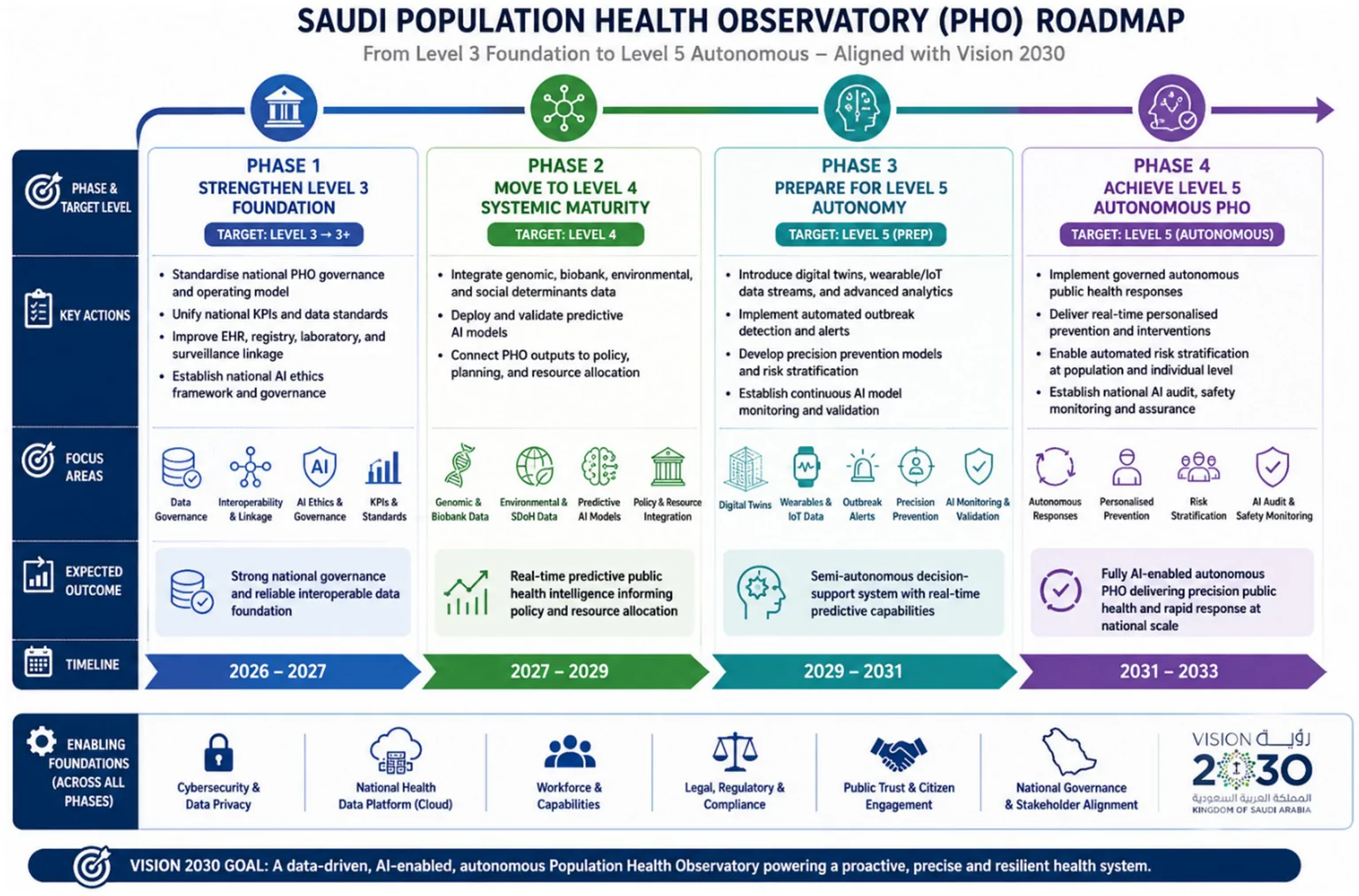
Illustrative policy scenario for progressive Saudi PHO capability development to advance from Level 3 to Level 5. The visual layout was prepared using generative AI; All substantive content was developed and verified by the authors.

**Figure 4:**
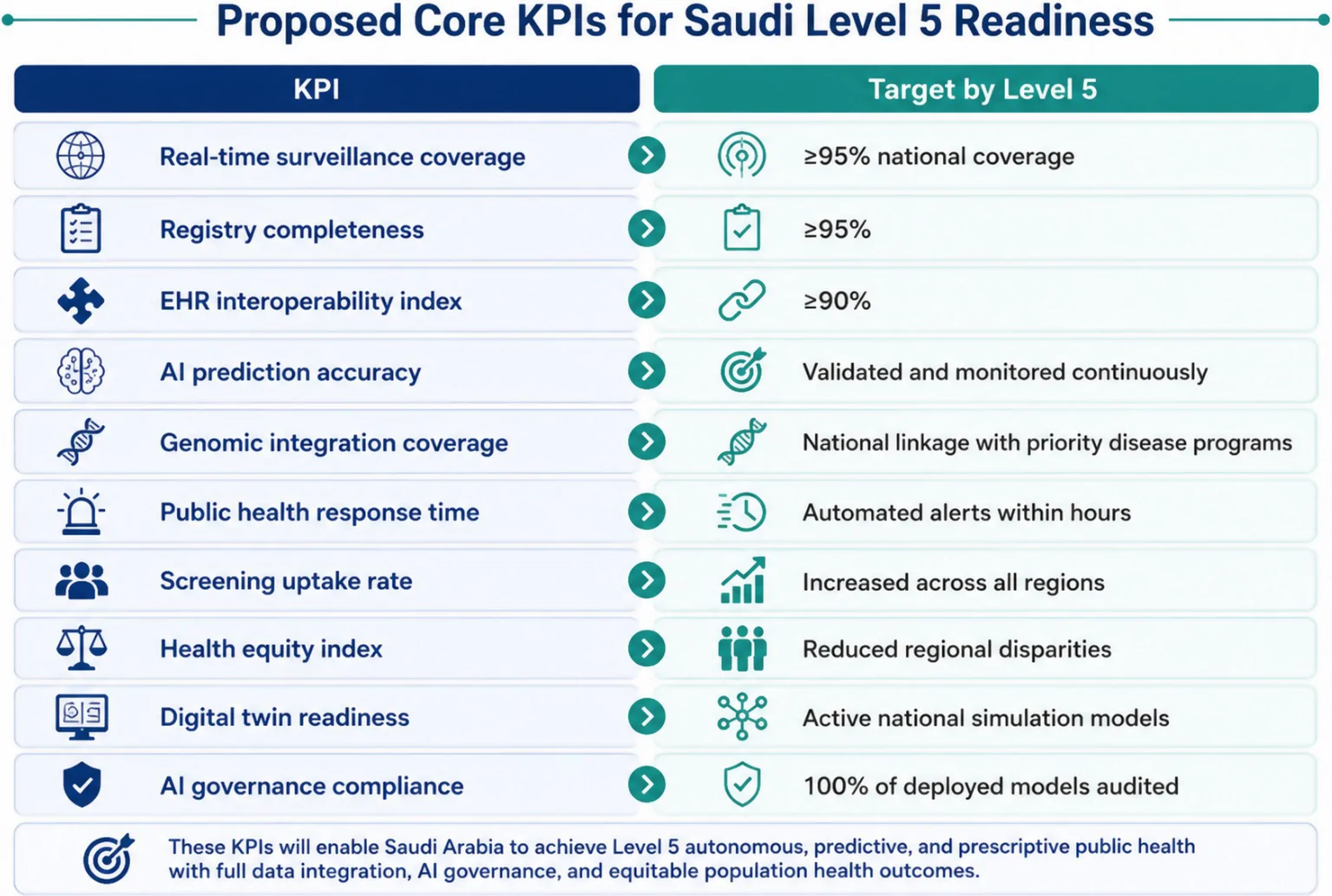
Proposed core key performance indicators for Saudi level 5 readiness.

Comparable implementation references are illustrative benchmarks from other health systems and are not direct cost or timeline estimates for Saudi Arabia, whose feasibility will depend on local infrastructure, governance, and resourcing.

## 4 Discussion

Saudi Arabia has made notable strides toward operational PHO maturity, driven by national digital transformation initiatives, registry development, and early adoption of AI in primary healthcare for diagnostics and clinical decision support (14, 49, 50), thereby enhancing patient outcomes, showing sensitivity and specificity rates between 82 and 97%, forecasting health risks through predictive models, and transforming healthcare delivery in alignment with Vision 2030. In addition, the government of Saudi Arabia managed to digitise 97% of public services (51). These efforts collectively aim to enhance healthcare efficiency, quality, accessibility, and service delivery (50, 52). By investigating global best practices relative to PHOs and comparing them to Saudi Arabia, this study revealed a clear vision toward evaluating PHOs for the advantage of national or global PHOs. The PHO provides an organisational platform that transforms fragmented contextual data, surveys, and SDoH into actionable interventions or policies (53–56). In this comparative research, differences in PHOs’ institutional structures were identified as consistently performing several core functions, including data interpretation and analysis, dissemination of dashboards and reports, indicator development, and support for evidence-based decision-making (6, 57, 58). The observatories were found to function through the Ministries of Health (14, 59–64), governmental agencies (12, 65) the Centres for Disease Control and Prevention (CDC) or other public health agencies (9–11, 66–70), regional collaborative networks (71–73) or academic institutions (74–80).

Modern PHOs have evolved beyond conventional disease surveillance, integrating geographic inequalities, SDoH, and fine-scale population comparisons into their decision-support and analytical frameworks (75, 80–82). Lessons from international experiences provide guidance for successful PHOs, which typically depend on clearly articulated strategic objectives, digital infrastructure, robust analytics, efficient data-sharing frameworks, and sustainable financing models, with transparent governance structures and accountability mechanisms (4, 5, 57, 71). Furthermore, collaborative interinstitutional networking has emerged as an essential driver for fundamental PHO effectiveness.

In particular, experiences from the UK and French observatory (see Supplementary Table 1) systems have determined that harmonised practices, pooled expertise, and coordinated collaborative networks advance analytical consistency, operational efficiency, and scalability across public health systems (55, 58). Similarly, global evaluations have underscored the growing importance of virtual expert networks and shared public health toolkits in supporting the development, sustainability, and standardisation of PHOs (57). Historically, the impact of PHOs is comparatively limited, with most evaluations primarily emphasising oriented process outcomes, such as stakeholder engagement, usability, dissemination effectiveness, and professional uptake, rather than measurable improvements in population-level health outcomes or indicators (83, 84). However, the current PHO models increasingly rely on interactive digital platforms and web-based dashboards, particularly within urban health surveillance and crisis-response or emergency settings and technologically enabled public health governance systems, reflecting the global transition toward real-time, data-driven public health governance (85–87).

### 4.1 Cross-national interpretation of maturity differences

Differences between countries with comparable income levels suggest that economic resources alone do not determine PHO maturity. Finland’s stronger profile relative to France may reflect Finland’s long-standing use of national registries, personal identifiers, centralised data-linkage infrastructure and established integration between health data, research and public administration. France has substantial public-health capacity, but its more distributed institutional architecture and heterogeneous data-governance arrangements may limit the visibility or uniformity of national integration across the variables assessed. Similarly, Singapore’s higher profile relative to Japan may reflect Singapore’s centralised governance, smaller population, integrated national digital-identity environment and capacity for rapid cross-agency implementation. Japan possesses highly advanced healthcare and research capabilities, but data fragmentation across providers, administrative structures and legacy information systems may constrain national-level interoperability. These interpretations remain tentative because the study relied on publicly available documentation and did not directly evaluate institutional processes within each country.

Saudi Arabia has made significant progress toward operational PHO maturity through its centralised public health systems, which provide strategically important transitions in national digital transformation. Since the establishment of its PHO in 2023, it has reached level 3 in terms of AI maturity. Its attainment of level 5, autonomous maturity, will require a strategically orchestrated transition from operational analytics toward real-time, fully interoperable, multicentred integration, which can support an AI-driven public health intelligence architecture that provides autonomous decision support. The immediate prerequisite for this transition is strengthening interoperability frameworks across EHRs, laboratory information systems, registries, genomic platforms, screening programs, and SDoH datasets. This approach should be followed by the systematic deployment of validated predictive and prescriptive analytics to support outbreak forecasting, disease burden modelling, precision prevention, and resource allocation. This integrated ecosystem must be complemented by the systematic deployment of rigorously validated predictive models by integrating biological markers and prescriptive analytical models designed to support dynamic forecasting for outbreaks, simulation of disease burden, optimised resource allocation, precision strategies for prevention, and prognosis and diagnoses to provide population-level risk stratification.

The present study represents an initial framework development and feasibility application rather than formal model validation. Subsequent validation should include multidisciplinary expert assessment of content relevance and completeness, calculation of item- and scale-level content-validity indices, pilot application by independent national assessors, evaluation of inter-rater and test–retest reliability, and examination of convergent or criterion validity against independent indicators of surveillance performance, data quality, policy responsiveness and population-health impact.

### 4.2 Limitations of the study

Although this study provides an overview of where we stand in Saudi Arabia and paves a futuristic road map for the PHO, several limitations of this study should be acknowledged, such as: First, the comparative assessment relied predominantly on publicly available documentation, reports, literature, and international databases. Consequently, countries with restricted public access or limited or not updated reports may have been underestimated despite the possibility of having advanced internal capabilities and operational capacities. Further, the purposive country-selection strategy may favour jurisdictions with stronger publication and transparency practices. Accordingly, higher scores may partly reflect documentation availability rather than superior operational capacity, whereas lower scores may reflect limited public reporting rather than true absence of capability. Moreover, a single standardised framework was applied across 21 countries with markedly different governance, legal, funding, and infrastructure contexts, without statistical or structural adjustment (e.g., stratification by system type or funding model). Rankings should be interpreted as relative/descriptive rather than fully context-adjusted comparative measures. This within-tier heterogeneity illustrates a broader pattern relevant to interpreting the framework’s rankings: smaller, centrally governed health systems may achieve faster, more uniform variable-level integration than larger, more devolved systems, even when both are ultimately classified at the same overall maturity level. Comparative maturity should therefore be read alongside system-level structural factors (population size, governance centralization, payer structure) rather than as a pure measure of technological sophistication.

Second, the study did not include direct engagement with the PHOs studied in this paper, nor a field-based validation; therefore, findings reflect a comprehensive operational assessment or independent audit of implementation based on documented maturity. Further to that, two authors were affiliated with the Saudi Ministry of Health, which oversees the PHO assessed in the case application. This creates a risk of institutional self-assessment bias, particularly in interpreting incomplete or favourable documentation. Unless independently rescored, the Saudi estimate should not be interpreted as an external audit or objective certification of maturity. Third, variation in publication dates influenced comparisons across countries, consequently affecting reporting practices, health system documents and the update frequency of national reports. Fourth, although predefined scoring evaluation criteria were systematically applied, the translation of qualitative-to-quantitative conversion scores inevitably involves a degree of subjective interpretative judgement. Fifth, and directly relevant to the interpretation of the composite score: all 18 maturity variables were weighted equally, meaning a foundational variable such as static dashboard reporting contributes identically to the composite score as domains such as AI governance, genomic integration, or digital twin capability. This is a significant methodological choice, not an incidental one, when calculating the composite AI maturity score. This approach assumes that domains such as interoperability, AI governance, predictive analytics, genomic integration, wearable/IoT data, digital twins, and population coverage contribute equally to overall maturity, an assumption made for methodological transparency and simplicity rather than one supported by empirical evidence or an established theoretical weighting model. It is plausible that certain variables (e.g., interoperability or AI governance) exert a disproportionately larger influence on functional maturity than others. Future iterations of this framework should explore alternative weighting strategies, such as expert-derived or Delphi-consensus weights, data-driven weighting through statistical methods (e.g., factor analysis or principal component analysis), or priority-based weighting aligned with national health strategies, and should conduct sensitivity analyses to assess the robustness of country maturity classifications and rankings under varying weighting schemes. Moreover, independent pre-consensus ratings were not retained in a form that permitted retrospective calculation of reliability. Consequently, the degree of independent concordance between raters, as distinct from the agreement achieved after discussion, cannot be quantified, which limits the ability to assess the objective reliability and reproducibility of the scoring process.

Sixth, the comparative analysis presented in this study is descriptive rather than inferential; no sensitivity analyses were conducted to test how rankings would change under alternative scoring or weighting assumptions, no uncertainty analyses (e.g., bootstrap resampling or confidence intervals) were performed to quantify the precision of composite scores, no clustering methods (e.g., k-means or hierarchical clustering) were applied to empirically verify whether countries form coherent maturity tiers rather than being assigned to categories *a priori*, and no external validation against independent benchmarks or objective performance data was undertaken; consequently, the reported differences between countries should be interpreted as an initial descriptive positioning rather than a statistically validated comparison. Finally, country inclusion was influenced by the availability and comprehensiveness of documentation with sufficient information related to PHO frameworks and digital health maturity, which may have introduced selection prejudice. Future investigations should incorporate direct stakeholder engagement, longitudinal evaluations and real-world implementation assessments to overcome the major limitation of the absence of interviews, field assessments and verification by national PHO representatives to monitor the continued evolution and the PHO global impact.

Given the current efforts to establish a robust AI governance ecosystem to ensure algorithmic transparency, explainability, ethical oversight, continuous model validation and mechanisms, this paper presents a futuristic roadmap to sustain public and institutional trust. Concurrently, through the integration of genomic surveillance, wearables, digital twin technologies and IoT-derived health data streams, continuously adaptive learning systems and a national biobanking observatory, through the consolidation of existing institutional biobank initiatives (e.g., Princess Nourah Bint Abdulrahman University, Public Health Authority and others) into a coordinated national biobanking observatory, which not only serves as a research accelerator but also for biological and population-driven decision making. This serves as both a research accelerator and a tool for biological and population-driven decision making. This will lead the Saudi PHO to evolve from operational maturity (Level 3) to autonomous intelligence maturity (Level 5). Such a transformation in the PHO would position Saudi Arabia at the forefront as a regional and global leader of precision public health, reduce regional health disparities, facilitate earlier disease detection, implement anticipatory and personalised prevention strategies, reduce regional and socioeconomic health inequities and enable highly responsive evidence-based policymaking aligned with the strategic objectives of Vision 2030.

## Conclusion

This study proposes a preliminary framework for describing the maturity of Population Health Observatories across governance, data integration, surveillance, analytical capability, policy translation, prevention and population outcomes. Its exploratory application identified substantial variation in publicly documented PHO capabilities, particularly in interoperability, real-time data exchange, predictive analytics and governance. However, the resulting profiles should not be interpreted as definitive national rankings because they are based on heterogeneous secondary documentation and an unvalidated scoring framework. The Saudi Arabian case illustrates how the framework may support structured identification of potential capability gaps, but it does not demonstrate that the proposed roadmap will improve health outcomes or establish national leadership. External stakeholder consultation, explicit scoring validation, independent country assessment and prospective evaluation against operational and population-health outcomes are required before the framework can be adopted for formal benchmarking or policy evaluation.

## Data Availability

The original contributions presented in the study are included in the article/Supplementary material, further inquiries can be directed to the corresponding author.
